# A novel posterior endoscopic cervical approach for treating cervical spondylotic radiculopathy: a finite-element analysis(C2-T1)

**DOI:** 10.3389/fbioe.2025.1623250

**Published:** 2025-10-28

**Authors:** Bo Lei, Chaofan Qin, Si Cheng, Qingshuai Yu, Jiming Liu, Xin Wang, Tao Hu, Ke Ma, Yu Chen, Zhengjian Yan

**Affiliations:** 1 Department of Spinal Surgery, The Second Affiliated Hospital of Chongqing Medical University, Chongqing, China; 2 ChongQing Breif Technology Co., Ltd. No. 1, Chongqing, China

**Keywords:** finite-element analysis, biomechanics, posterior endoscopic cervical discectomy, radiculopathy, safety

## Abstract

**Objective:**

This study aimed to preliminarily demonstrate the safety of using a novel surgical approach and investigate the biomechanical effects of different surgical approaches on the cervical spine.

**Methods:**

A finite-element model of an intact C2–T1 cervical spine was established. Different posterior endoscopic cervical discectomy (PECD) surgical approach models were constructed based on the intact model. The T1 inferior end was fully fixed, and a 100-N compressive load was applied to the odontoid process to simulate the head weight. A 2.0 Nm moment was applied to the odontoid process in three anatomical planes to simulate flexion-extension, lateral bending toward the surgical side, and rotation toward the surgical side. The range of motion (ROM), C6 pedicle stress, C6 facet joint stress, and intervertebral disk stress were calculated under different loading conditions.

**Results:**

The finite-element simulations revealed that 1. Conventional PECD resulted in ROM changes within 5%, while the novel approach led to ROM variations depending on the bone tunnel preparation and motion type, with a maximum increase of 7.4%. 2. The novel approach altered C6 pedicle stress, with peak stress reaching 66.6 MPa (5.4 times the normal maximum), whereas conventional PECD had negligible effects on pedicle stress. 3. Conventional l PECD increased facet joint stress by up to 10.2%, whereas the novel approach changed it within 6.6%. 4. Both approaches caused less than a 5% change in intervertebral disk pressure.

**Conclusion:**

This study preliminarily demonstrates that the novel surgical approach is safe, with daily activity loads unlikely to cause fractures in the lateral mass or pedicle. Compared to the intact model, neither approach significantly affected the cervical ROM or disk pressure. Additionally, the novel approach had a lesser impact on facet joints, suggesting it may be a potentially advantageous option for PECD. Based on the pedicle stress and ROM changes, preserving the inferomedial quarter of the pedicle is beneficial, and minimizing structural disruption while effectively decompressing the nerve root should be prioritized.

## 1 Introduction

Cervical spondylosis is a widespread health concern globally (Safiri et al., 2020). Epidemiological studies indicate that cervical radiculopathy (CR) predominates among patients seeking medical care in China (Baojian et al., 2022). While conservative management may alleviate symptoms in some CR cases, surgical intervention provides rapid symptomatic relief (Bono et al., 2011). For patients refractory to conservative treatments, surgery often remains the sole therapeutic option (Bono et al., 2011).

Regarding surgical approaches, substantial evidence confirms that posterior endoscopic cervical discectomy (PECD) is an effective treatment for CR, demonstrating comparable clinical efficacy to anterior cervical discectomy and fusion (ACDF) (Lv et al., 2022; Zhang et al., 2022; Ahn, 2023). However, the conventional PECD via Key-hole approach (PECD-KH) requires access through the “V-point” (the intersection of the superior/inferior lamina and facet joint (Zhong et al., 2022)) to establish the working tunnel. This technique inevitably causes iatrogenic damage to the facet joint, potentially leading to postoperative instability (Zdeblick et al., 1992; Chang et al., 2023). Studies report a 4.9% incidence of cervical instability following PECD-KH (Lee et al., 2017; Jagannathan et al., 2009).

To minimize facet joint disruption, our institution has pioneered several modified PECD techniques, including translaminar foraminotomy approach (Liu et al., 2019), “trench technique (Yu et al., 2019),” and lateral mass-based PECD (PECD-LM) (Chen et al., 2024). The PECD-LM approach initiates the bone tunnel within the cervical lateral mass, accessing the spinal canal by partial resection of the lateral mass and pedicle—preserving facet joint integrity (Chen et al., 2024). Although preliminary studies suggest PECD-LM is safe and effective (Chen et al., 2024), limited follow-up durations preclude the possibility of definitive conclusions about its long-term safety.

Finite-element analysis (FEA), a well-established biomechanical research tool (Karpiński et al., 2016; Sun et al., 2024; Lin et al., 2024; Moldovan et al., 2019), was employed in this study to (1) Simulate the biomechanical impact of different bone tunnel configurations. (2) Predict the safety profiles of respective surgical approaches.

## 2 Materials and methods

### 2.1 Construction of the C2–T1 finite-element baseline model

**FIGURE 1:**
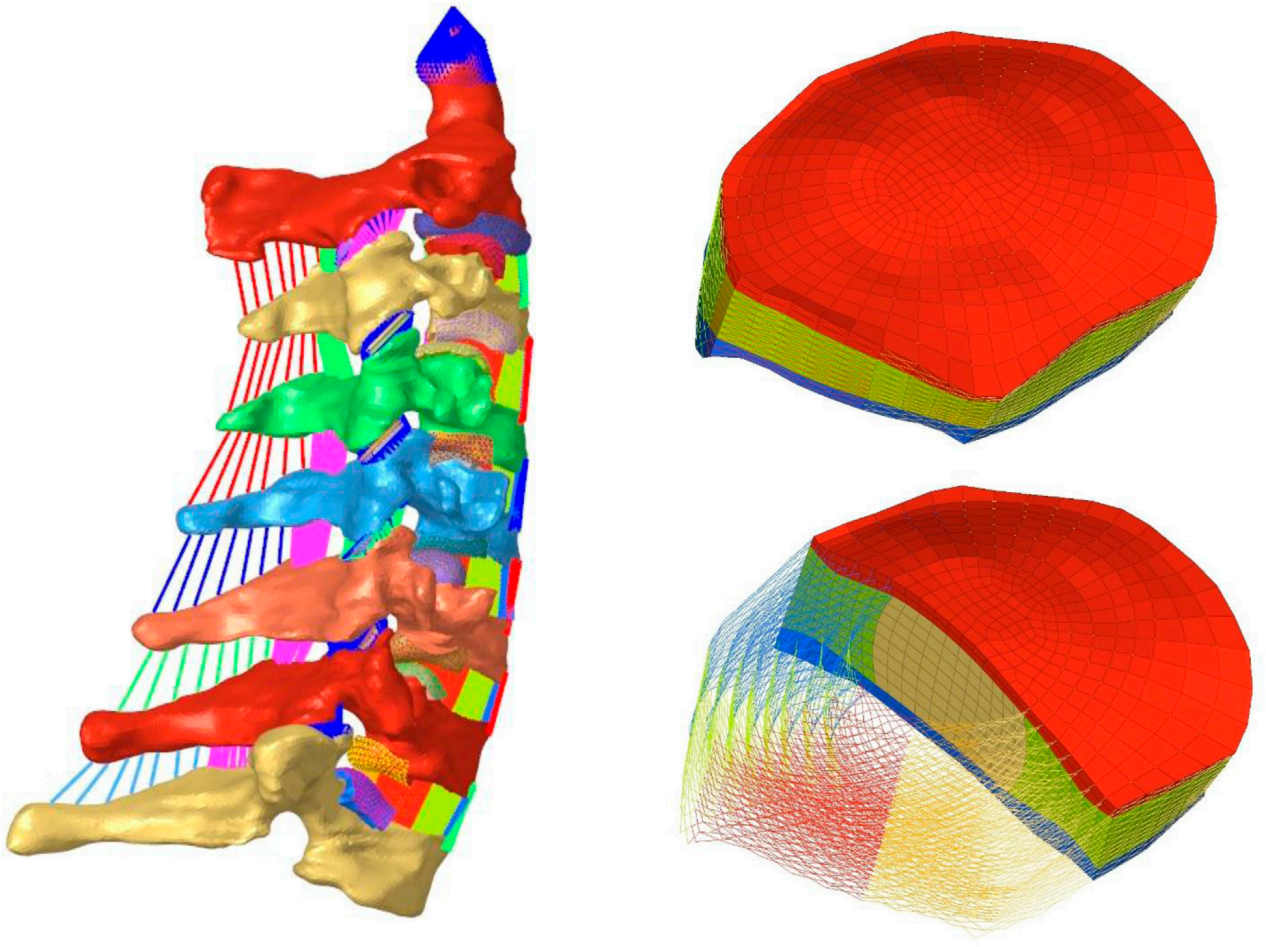
Finite-element model of intact C2–T1 and components (MO).

The validated cervical spine finite-element model (FEM) was adopted as the intact model (Wang et al., 2016; Wang et al., 2017) (M0, Figure 1). This model was derived from a “normal” 22-year-old female cadaveric specimen, encompassing the C2–T1 segments, and constructed following the methodology described by Wang et al. (2016). The finite-element modeling methodology has been comprehensively detailed in the works of Wang et al.; therefore, these technical aspects will not be redundantly addressed in the present study. For specific modeling parameters and implementation details, refer Wang et al. (2016). Material properties of all components are summarized in Table 1.

**TABLE 1:** Material properties of model components.

Name	Element type	Material model	Material property	References
Cancellous bone	C3D4	ISO elastic	E = 300 MPa μ = 0.3	Safiri et al. (2020)
Cortical bone	C3D4	ISO elastic	E = 12000 MP μ = 0.3	Safiri et al. (2020)
Cartilaginous end-plate	C3D8	ISO elastic	E = 23.8 MPa μ = 0.3	Baojian et al. (2022)
Cartilage of joint	C3D8	ISO elastic	E = 23.8 MPa μ = 0.3	Baojian et al. (2022)
Nucleus	C3D8H	Hyperelastic	C10 = 0.12, C01 = 0.09	Baojian et al. (2022)
Annulus ground substance	C3D8H	Hyperelastic	C10 = 0.133, C01 = 0.0333, D=0.6	Bono et al. (2011), Lv et al. (2022)
Annulus fiber	SpringA	Nonlinear spring	Stress–strain curve	Zhang et al. (2022), Ahn (2023), Baojian et al. (2022)
Ligaments	SpringA	Nonlinear spring	Force–defection curve	Zhong et al. (2022), Zdeblick et al. (1992)

E, Young’s modulus; μ, Poisson’s ratio; Cij, D material constant; ISO elastic, isotropic.

### 2.2 Surgical modeling of the C5/6 segment

**FIGURE 2:**
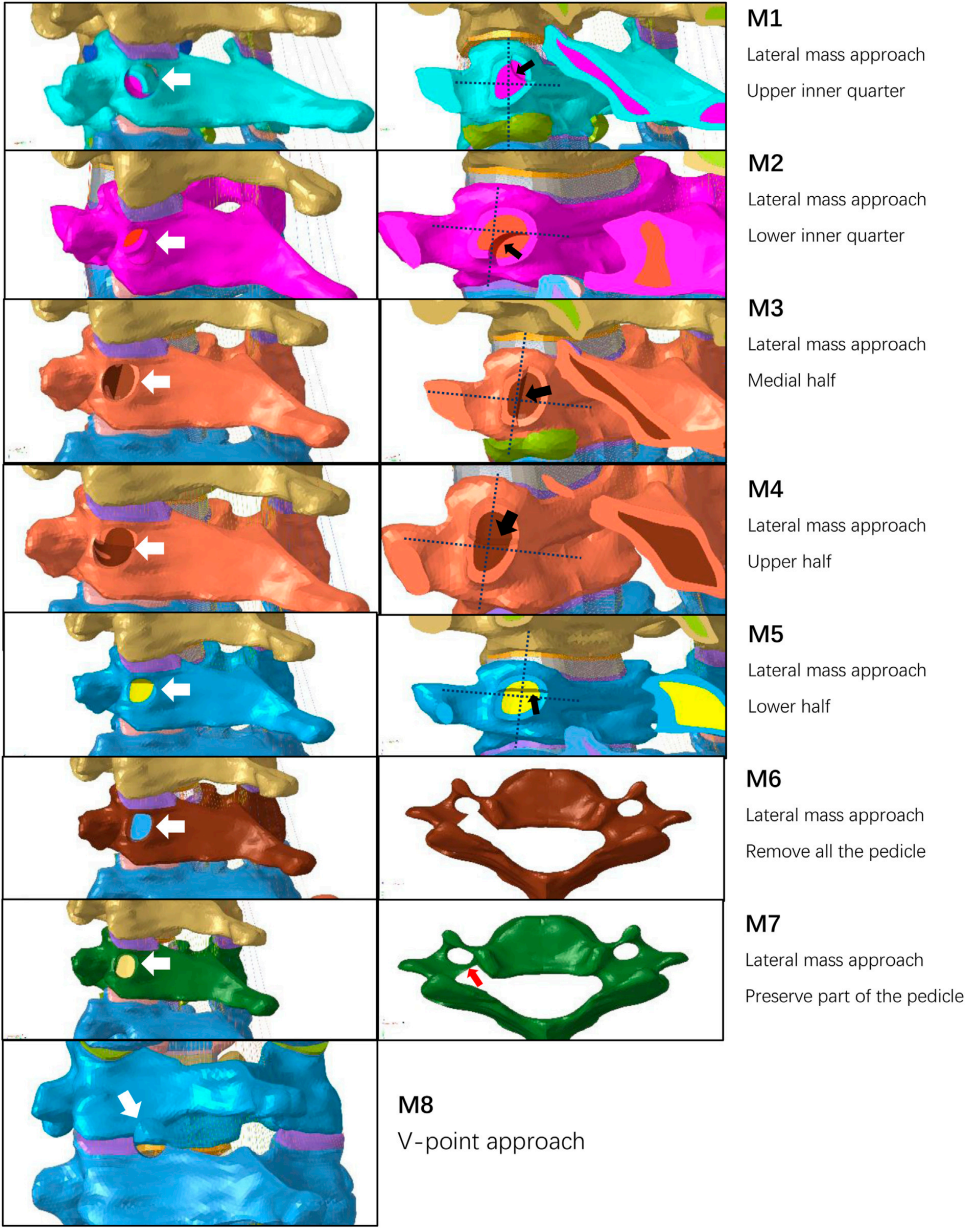
Schematic diagram of the M1–M8 surgical model. (A1–A7) Schematic diagram of the bone defect on the lateral mass surface, the white arrow points to the bone tunnel. (B1–B5) Schematic diagram of the resected area of the pedicle, the dashed line divides the coronal plane of the pedicle into four parts, and the black arrow points to the portion of the pedicle that has been resected. (C1 and C2) Top view of the C6 vertebra; the red arrow points to the remaining pedicle. (D) Schematic diagram of the V-point approach with a resection of less than 50% of the facet joint.

To simulate surgical bone tunnels, a 6-mm-diameter defect was created at the left C5/6 level, with tunnel origins at the C6 lateral mass for PECD-LM and the V-point for PECD-KH (Figure 2). Eight surgery models (M1–M8) were established: M1 (lateral mass approach with superomedial pedicle quadrant damage), M2 (inferomedial pedicle quadrant damage), M3 (medial half pedicle resection), M4 (superior half pedicle resection), M5 (inferior half pedicle resection), M6 (complete pedicle resection), M7 (lateral pedicle cortex preservation only), and M8 (a resection area of the facet joint less than 50%).

### 2.3 Loading and boundary conditions

The T1 inferior end was fully fixed, and a compressive load of 100 N—the maximum value reported in previous studies (e.g., 50 N (Du et al., 2024; He et al., 2021), 73.6 N (Lin et al., 2024), and 100 N (Wang et al., 2019; Li et al., 2024))—was applied to the apex of the dens to simulate the head weight, ensuring a rigorous safety assessment. Moments of 2.0 Nm (the highest among commonly used values such as 1.0 Nm (Lin et al., 2024), 1.5 Nm (Huang et al., 2023), and 2.0 Nm (Wang et al., 2016)) were applied to the dens in three anatomical planes to simulate flexion–extension, lateral bending toward the surgical side, and axial rotation toward the surgical side, further validating biomechanical safety under extreme conditions.

## 3 Results

### 3.1 Validation of finite-element models

**FIGURE 3:**
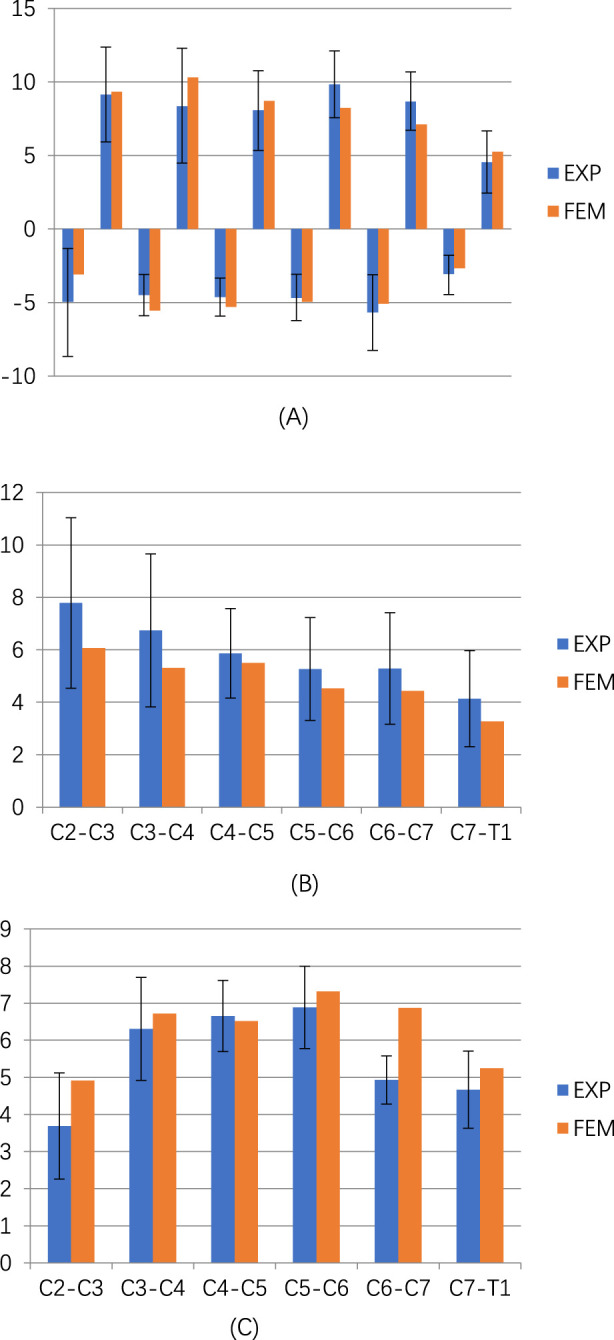
Comparison of the ROM of the intact three-dimensional finite-element models of C2–T1 with the prior biomechanical studies. **(A)** ROM in flexion-extension. **(B)** ROM in lateral bending. **(C)** ROM in axial rotation.

The intact model (M0) in this study had been previously validated in earlier research (Wang et al., 2016; Wang et al., 2017). Since the loading and boundary conditions differed from prior studies, we again compared the ROM values of each segment in the M0 model with those reported in the literature. The results demonstrated a strong agreement between the average ROM values in this study and those in existing publications (Wheeldon et al., 2006; Yoganandan et al., 2007; Yoganandan et al., 2008; Figure 3), confirming the reliability and accuracy of the M0 model.

### 3.2 ROM changes across different movement modes

**FIGURE 4:**
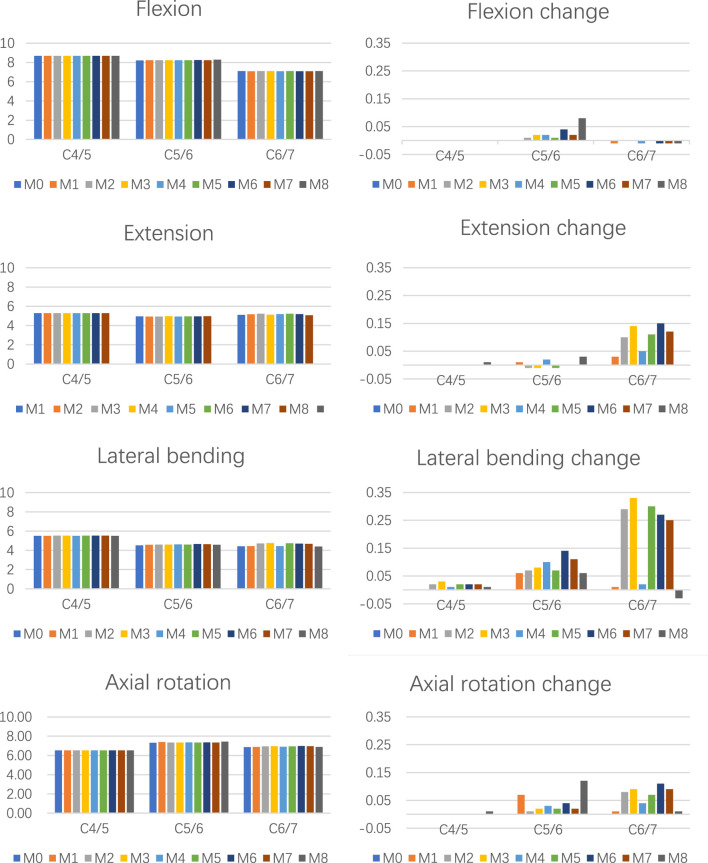
The ROM and △ROM for C4/5–C6/7 segment among different groups during various movements.

Compared to the intact model (M0), the ROM values in groups M1–M8 showed no significant changes during flexion, extension, or axial rotation. The maximum ROM variation was less than 1% in flexion, under 3% in extension, and within 5% in axial rotation (Figure 4). During lateral bending, the M8 model exhibited nearly no change relative to M0, while M1–M7 displayed the following characteristics: the C4/5 segment ROM remained almost unchanged, the C5/6 segment showed minor alterations, and the C6/7 segment demonstrated the most pronounced changes. At C6/7, most of the novel surgical approaches (except M1 and M4) significantly increased ROM, with the maximum change observed in M3 (0.33°, 7.4% increase). In contrast, M1 and M4 models exhibited changes of less than 3%.

### 3.3 Changes in C6 pedicle stress

**FIGURE 5:**
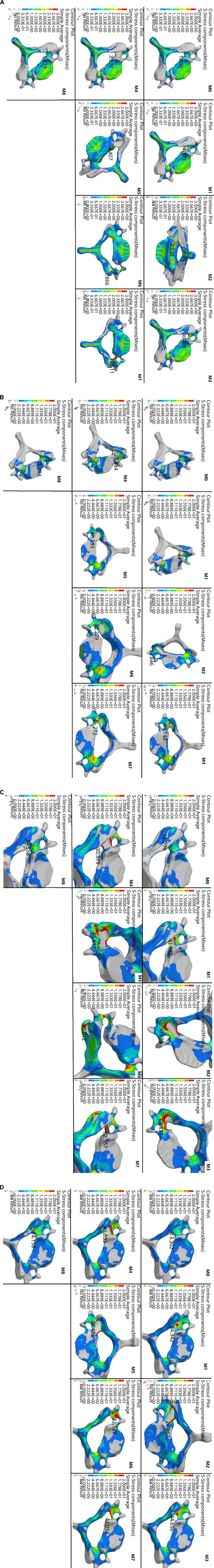
**(A)** C6 pedicle stress during flexion (MPa). **(B)** C6 pedicle stress during extension (MPa). **(C)** C6 pedicle stress during lateral bending (MPa). **(D)** C6 pedicle stress during axial rotation (MPa).

The stress distribution in the C6 pedicle was notably influenced by surgical approaches (Figures 5A–D). The M8 model showed a minimal impact, with stress patterns closely resembling those of M0 across all motion modes. In the M6 model, complete pedicle resection altered the stress transmission path, leading to stress concentration in the ipsilateral transverse process—although peak stress did not exceed 45 MPa. In M1–M7 (excluding M6), pedicle stress increased substantially (peak stresses exceeding twice those of M0), with the most significant changes in M3, M5, and M7, and the least in M4. Among all motion modes, pedicle stress exceeded 60 MPa only during lateral bending, occurring in M3, M5, and M7.

### 3.4 Alterations in C6 facet joint stress

**FIGURE 6:**
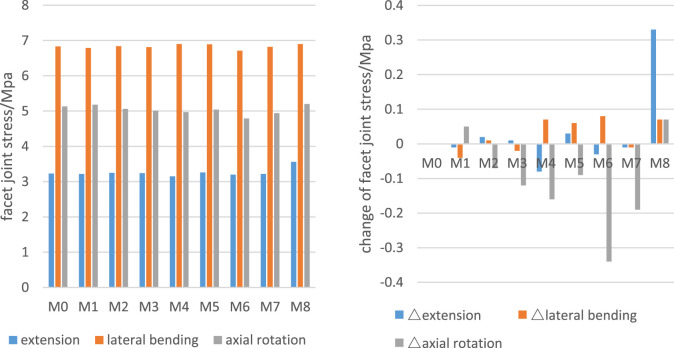
C6 facet joint stress and changes of the number.

Figure 6 presents the C6 facet joint stress among different groups during various movements. All models exhibited varying degrees of facet joint stress alterations at C6. The M8 model demonstrated consistent stress increases across motion modes, particularly during extension where stresses increased by 0.33 MPa (10.7% increase). In M1–M7 models, facet joint stress changes were generally insignificant, except in M6 during axial rotation, where a 6.63% stress reduction was observed (the only instance >5% variation among these models).

### 3.5 Intervertebral disk pressure (IDP)

**FIGURE 7:**
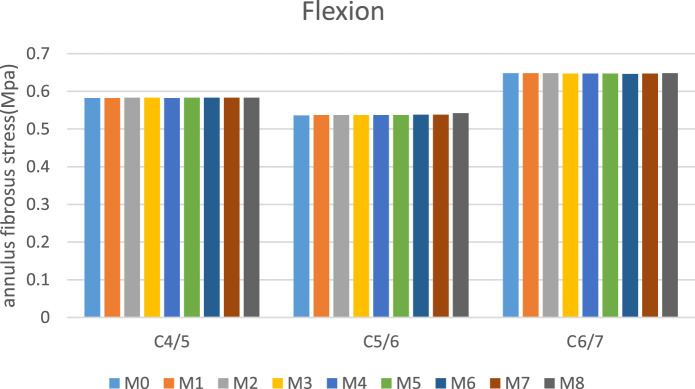
C5/6 nucleus pulposus stress and annulus fibrosus stress.

Figure 7 presents the IDP of C5/6 among different groups during various movements. Across all motion modes, the maximum stresses in both the nucleus pulposus and annulus fibrosus of intervertebral disks demonstrated negligible changes (<5%) in surgical models M1–M8 compared to M0, indicating minimal biomechanical impact on disk loading characteristics.

## 4 Discussion

The key-hole approach has been widely adopted in both clinical practice and scientific research (He et al., 2021; Yuchi et al., 2019; Ke et al., 2020). Our modified surgical approach, developed from this established technique, preserves the advantages of minimally invasive surgery while significantly reducing the impact on cervical facet joints. This innovation maintains surgical safety and demonstrates superior biomechanical preservation compared to conventional methods.

### 4.1 Changes of ROM

Alterations in cervical ROM are considered associated with cervical degeneration (Bogduk and Mercer, 2000; Gore et al., 1986). Compared to fusion surgery, PECD has a lesser impact on cervical ROM, which is regarded as one of its advantages. Previous studies have shown that conventional PECD approaches affect ROM in both the surgical and adjacent segments, with the most pronounced changes (up to 20% (He et al., 2021; Ke et al., 2020)) occurring at the surgical level. In this study, PECD-KH demonstrated a negligible influence on cervical ROM, consistent with findings by He et al. (2021). Discrepancies between studies may stem from differences in bone tunnel size (Choi et al., 2024), facet joint resection extent (Choi et al., 2024), or variations in intact model parameters, loading conditions, and boundary constraints.

This study revealed two characteristic patterns of cervical ROM alterations: (1) In the PECD-LM group, the most significant ROM change occurred at C6/7 (the subjacent segment), whereas PECD-KH showed no notable alterations at any level. This suggests that while PECD-LM minimally affects the surgical segment, it may induce slight compensatory hypermobility in the subjacent segment, possibly due to factors like elastic deformation of the bone tunnel. (2) Post-PECD-LM, ROM changes were motion-dependent: negligible during flexion-extension and rotation but more pronounced during lateral bending. This implies that the pedicle may contribute to cervical stability during lateral bending, and long-term lateral bending postoperatively could accelerate degeneration.

### 4.2 C6 pedicle stress

Few studies have examined PECD’s effect on pedicle stress, with prior focus on ROM, disk stress, facet joint stress, and uncovertebral joint stress (He et al., 2021). PECD-LM, by disrupting the lateral mass and pedicle, altered the C6 stress distribution, whereas PECD-KH showed minimal impact. Stress changes were most evident during lateral bending and rotation, with flexion-extension causing smaller variations. Among all models, M1–M7 (excluding M6) exhibited peak pedicle stress during lateral bending; M6 (complete pedicle resection) shifted stress concentration to the transverse foramen and lateral mass surface.

Concerns about stress-induced fractures are reasonable, particularly given elevated stresses in some models during lateral bending. However, cortical bone’s axial compressive (170 MPa (Rho et al., 1998)) and tensile (130 MPa (Reilly and Burstein, 1975)) strengths far exceed the peak stress in this study (66.6 MPa), confirming PECD-LM’s safety regarding bone tunnel integrity.

### 4.3 C6 facet joint stress

Increased facet joint stress has been identified as a potential risk factor for cervical degeneration (He et al., 2021). While the key-hole technique consistently elevated facet joint stresses across all motion modes (with near 10% increases during extension), the novel surgical approach demonstrated no significant stress augmentation due to its facet sparing nature. These findings suggest that PECD-LM may potentially decelerate degenerative progression compared to PECD-KH.

### 4.4 IDP

Both the current study and prior investigations consistently demonstrate that PECD-KH induces minimal alterations in IDP (Yuchi et al., 2019; Ke et al., 2020). Notably, our findings further indicate that PECD-LM similarly preserves this advantageous characteristic.

### 4.5 Intra-model comparison: PECD-LM

Models M1 and M4 outperformed others in ROM and pedicle stress preservation: (1) ROM changes were minimal (max 2.2% at C5/6 during lateral bending in M4). (2) Pedicle stress changes were least pronounced. These models uniquely preserved the inferomedial pedicle quarter, suggesting this region’s biomechanical significance—a hypothesis requiring further validation, as no literature addresses segmental pedicle roles.

### 4.6 Limitations

This study has several limitations: (1) The finite-element model represents a simplified simulation that cannot fully replicate *in vivo* cervical biomechanics, particularly as it omits the musculature that contributes to spinal stability; however, since both PECD-KH and PECD-LM share this limitation comparably, the comparative results remain valid. (2) The analysis focused solely on surgical access effects without simulating complete PECD procedures involving posterior longitudinal ligament, intervertebral disk, or uncovertebral joint modifications—factors that may influence postoperative biomechanics more significantly than bone tunnels alone; nevertheless, as both techniques produce similar effects on these structures, the observed differences primarily reflect tunnel-related variations, though future studies will incorporate postoperative models for comprehensive assessment. (3) The model was derived from imaging data of a single healthy young woman, lacking degenerative features typical of cervical radiculopathy; however, since the bone tunnels primarily involve the lateral mass and pedicle (structures often preserved in such patients), the findings retain relevance, pending validation in broader demographic cohorts including varied ages, genders, and pathological conditions. (4) While the study employed high mechanical loads to test safety margins, conclusions may not generalize to patients with severe comorbidities like osteoporosis, where physiological loads could risk pedicle fracture despite the model’s safety thresholds.

## 5 Conclusion

This study preliminarily demonstrates that the novel surgical approach is safe, with daily activity loads unlikely to cause fractures in the lateral mass or pedicle. Compared to the intact model, neither approach significantly affected cervical ROM or disk pressure. Additionally, the novel approach had a lesser impact on facet joints, suggesting it may be a potentially advantageous option for PECD. Based on pedicle stress and ROM changes, preserving the inferomedial quarter of the pedicle is beneficial, and minimizing structural disruption while effectively decompressing the nerve root should be prioritized.

## Data Availability

The original contributions presented in the study are included in the article/Supplementary Material; further inquiries can be directed to the corresponding author.

## References

[B1] Ahn Y. (2023). Anterior endoscopic cervical discectomy: surgical technique and literature review. Neurospine 20 (1), 11–18. 10.14245/ns.2346118.059 37016849 PMC10080429

[B2] Baojian W. Junhai L. Hu H. Jinghua G. Zhaojun C. Dong Y. (2022). Analysis on the clinical epidemiological characteristics of outpatients with cervical spondylosis in a three a and tertiary hospital from 2018 to 2020 in beijing. Chin. Med. Rec. 23 (12), 40–43.

[B3] Bogduk N. Mercer S. (2000). Biomechanics of the cervical spine. I: normal kinematics. Clin. Biomech. (Bristol) 15 (9), 633–648. 10.1016/s0268-0033(00)00034-6 10946096

[B4] Bono C. M. Ghiselli G. Gilbert T. J. Kreiner D. S. Reitman C. Summers J. T. (2011). An evidence-based clinical guideline for the diagnosis and treatment of cervical radiculopathy from degenerative disorders. Spine J. 11 (1), 64–72. 10.1016/j.spinee.2010.10.023 21168100

[B5] Chang C. J. Liu Y. F. Hsiao Y. M. Chang W. L. Hsu C. C. Liu K. C. (2023). Full endoscopic spine surgery for cervical spondylotic myelopathy: a systematic review. World Neurosurg. 175, 142–150. 10.1016/j.wneu.2023.05.012 37169077

[B6] Chen M. Yu Q. Cheng S. Hu T. Wang X. Lei B. (2024). Posterior lateral endoscopic cervical discectomy through a lateral mass approach in the treatment of cervical spondylotic radiculopathy. World Neurosurg. 185, e1064–e1073. 10.1016/j.wneu.2024.03.024 38490445

[B7] Choi H. Purushothaman Y. Ozobu I. Yoganandan N. (2024). Is posterior cervical foraminotomy better than fusion for warfighters? a biomechanical study. Mil. Med. 189 (Suppl. 3), 710–718. 10.1093/milmed/usae235 39160815

[B8] Du Q. Wang Z. J. Zheng H. D. Wang S. F. Cao G. R. Xin Z. J. (2024). Anterior percutaneous full-endoscopic transcorporeal decompression for cervical disc herniation: a finite element analysis and long-term follow-up study. BMC Musculoskelet. Disord. 25 (1), 639. 10.1186/s12891-024-07754-x 39134982 PMC11321056

[B9] Gore D. R. Sepic S. B. Gardner G. M. (1986). Roentgenographic findings of the cervical spine in asymptomatic people. Spine (Phila Pa 1976) 11 (6), 521–524. 10.1097/00007632-198607000-00003 3787320

[B10] He T. Zhang J. Yu T. Wu J. Yuan T. Liu R. (2021). Comparative analysis of the biomechanical characteristics after different minimally invasive surgeries for cervical spondylopathy: a finite element analysis. Front. Bioeng. Biotechnol. 9, 772853. 10.3389/fbioe.2021.772853 34976969 PMC8716838

[B11] Huang S. Ling Q. Lin X. Qin H. Luo X. Huang W. (2023). Biomechanical evaluation of a novel anterior transpedicular screw-plate system for anterior cervical corpectomy and fusion (accf): a finite element analysis. Front. Bioeng. Biotechnol. 11, 1260204. 10.3389/fbioe.2023.1260204 38026869 PMC10665523

[B12] Jagannathan J. Sherman J. H. Szabo T. Shaffrey C. I. Jane J. A. (2009). The posterior cervical foraminotomy in the treatment of cervical disc/osteophyte disease: a single-surgeon experience with a minimum of 5 years' clinical and radiographic follow-up. J. Neurosurg. Spine 10 (4), 347–356. 10.3171/2008.12.spine08576 19441994

[B13] Karpiński R. Jaworski Ł. Zubrzycki J. (2016). Structural analysis of articular cartilage of the hip joint using fini. Adv. Sci. Technol. Res. J. 10 (31), 240–246. 10.12913/22998624/64064

[B14] Ke W. Zhi J. Hua W. Wang B. Lu S. Fan L. (2020). Percutaneous posterior full-endoscopic cervical foraminotomy and discectomy: a finite element analysis and radiological assessment. Comput. Methods Biomech. Biomed. Engin 23 (12), 805–814. 10.1080/10255842.2020.1765162 32406769

[B15] Lee Y. S. Kim Y. B. Park S. W. Kang D. H. (2017). Preservation of motion at the surgical level after minimally invasive posterior cervical foraminotomy. J. Korean Neurosurg. Soc. 60 (4), 433–440. 10.3340/jkns.2015.0909.006 28689392 PMC5544370

[B16] Li K. Yu Q. Wang C. Zhang R. Fu Q. Feng Y. (2024). Biomechanical study of the stability of posterior cervical expansive open-door laminoplasty combined with bilateral c4/5 foraminotomy and short-segment lateral mass screw fixation: a finite element analysis. J. Orthop. Surg. Res. 19 (1), 620. 10.1186/s13018-024-05050-x 39363204 PMC11448283

[B17] Lin Z. Lin D. Xu L. Chen Q. Vashisth M. K. Huang X. (2024). Biomechanical evaluation on a new type of vertebral titanium porous mini-plate and mechanical comparison between cervical open-door laminoplasty and laminectomy: a finite element analysis. Front. Bioeng. Biotechnol. 12, 1353797. 10.3389/fbioe.2024.1353797 38375455 PMC10875091

[B18] Liu C. Liu K. Chu L. Chen L. Deng Z. (2019). Posterior percutaneous endoscopic cervical discectomy through lamina-hole approach for cervical intervertebral disc herniation. Int. J. Neurosci. 129 (7), 627–634. 10.1080/00207454.2018.1503176 30238849

[B19] Lv J. Mei J. Feng X. Tian X. Sun L. (2022). Clinical efficacy and safety of posterior minimally invasive surgery in cervical spondylosis: a systematic review. J. Orthop. Surg. Res. 17 (1), 389. 10.1186/s13018-022-03274-3 35964065 PMC9375334

[B20] Moldovan L. Gligor A. Grif H. S. Moldovan F. (2019). Dynamic numerical simulation of the 6-pgk parallel robot manipulator.

[B21] Reilly D. T. Burstein A. H. (1975). The elastic and ultimate properties of compact bone tissue. J. Biomech. 8 (6), 393–405. 10.1016/0021-9290(75)90075-5 1206042

[B22] Rho J. Y. Kuhn-Spearing L. Zioupos P. (1998). Mechanical properties and the hierarchical structure of bone. Med. Eng. Phys. 20 (2), 92–102. 10.1016/s1350-4533(98)00007-1 9679227

[B23] Safiri S. Kolahi A. A. Hoy D. Buchbinder R. Mansournia M. A. Bettampadi D. (2020). Global, regional, and national burden of neck pain in the general population, 1990-2017: systematic analysis of the global burden of disease study 2017. Bmj 368, m791. 10.1136/bmj.m791 32217608 PMC7249252

[B24] Sun X. Huang J. Zhang Q. Cao L. Liu Y. Song Z. (2024). Segment selection for fusion and artificial disc replacement in the hybrid surgical treatment of noncontiguous cervical spondylosis: a finite element analysis. Front. Bioeng. Biotechnol. 12, 1345319. 10.3389/fbioe.2024.1345319 38633668 PMC11021715

[B25] Wang Z. Zhao H. Liu J. M. Tan L. w. Liu P. Zhao J. h. (2016). Resection or degeneration of uncovertebral joints altered the segmental kinematics and load-sharing pattern of subaxial cervical spine: a biomechanical investigation using a c2-t1 finite element model. J. Biomech. 49 (13), 2854–2862. 10.1016/j.jbiomech.2016.06.027 27457429

[B26] Wang Z. Zhao H. Liu J. M. Chao R. Chen T. b. Tan L. w. (2017). Biomechanics of anterior plating failure in treating distractive flexion injury in the caudal subaxial cervical spine. Clin. Biomech. (Bristol, Avon) 50, 130–138. 10.1016/j.clinbiomech.2017.10.017 29100186

[B27] Wang X. D. Feng M. S. Hu Y. C. (2019). Establishment and finite element analysis of a three-dimensional dynamic model of upper cervical spine instability. Orthop. Surg. 11 (3), 500–509. 10.1111/os.12474 31243925 PMC6595113

[B28] Wheeldon J. A. Pintar F. A. Knowles S. Yoganandan N. (2006). Experimental flexion/extension data corridors for validation of finite element models of the young, normal cervical spine. J. Biomechanics 39 (2), 375–380. 10.1016/j.jbiomech.2004.11.014 16321642

[B29] Yoganandan N. Pintar F. A. Stemper B. D. Wolfla C. E. Shender B. S. Paskoff G. (2007). Level-dependent coronal and axial moment-rotation corridors of degeneration-free cervical spines in lateral flexion. JBJS 89 (5), 1066–1074. 10.2106/00004623-200705000-00020 17473145

[B30] Yoganandan N. Stemper B. D. Pintar F. A. Baisden J. L. Shender B. S. Paskoff G. (2008). Normative segment-specific axial and coronal angulation corridors of subaxial cervical column in axial rotation. Spine 33 (5), 490–496. 10.1097/brs.0b013e3181657f67 18317191

[B31] Yu K. X. Chu L. Chen L. Shi L. Deng Z. L. (2019). A novel posterior trench approach involving percutaneous endoscopic cervical discectomy for central cervical intervertebral disc herniation. Clin. Spine Surg. 32 (1), 10–17. 10.1097/bsd.0000000000000680 29979215

[B32] Yuchi C. X. Sun G. Chen C. Liu G. Zhao D. Yang H. (2019). Comparison of the biomechanical changes after percutaneous full-endoscopic anterior cervical discectomy versus posterior cervical foraminotomy at c5-c6: a finite element-based study. World Neurosurg. 128, e905–e911. 10.1016/j.wneu.2019.05.025 31096026

[B33] Zdeblick T. A. Zou D. Warden K. E. McCabe R. Kunz D. Vanderby R. (1992). Cervical stability after foraminotomy. A biomechanical *in vitro* analysis. J. Bone Jt. Surg. Am. 74 (1), 22–27. 10.2106/00004623-199274010-00004 1734010

[B34] Zhang J. Zhou Q. Yan Y. Ren J. Wei S. Zhu H. (2022). Efficacy and safety of percutaneous endoscopic cervical discectomy for cervical disc herniation: a systematic review and meta-analysis. J. Orthop. Surg. Res. 17 (1), 519. 10.1186/s13018-022-03365-1 36456964 PMC9714009

[B35] Zhong G. Feng F. Su X. Chen X. Zhao J. Shen H. (2022). Minimally invasive full-endoscopic posterior cervical foraminotomy and discectomy: introducing a simple and useful localization technique of the v point. Orthop. Surg. 14 (10), 2625–2632. 10.1111/os.13476 36102205 PMC9531083

