# Correction: A novel posterior endoscopic cervical approach for treating cervical spondylotic radiculopathy: a finite-element analysis(C2-T1)

**DOI:** 10.3389/fbioe.2025.1750232

**Published:** 2025-12-09

**Authors:** Bo Lei, Chaofan Qin, Si Cheng, Qingshuai Yu, Jiming Liu, Xin Wang, Tao Hu, Ke Ma, Yu Chen, Zhengjian Yan

**Affiliations:** 1 Department of Spinal Surgery, The Second Affiliated Hospital of Chongqing Medical University, Chongqing, China; 2 ChongQing Breif Technology Co., Ltd. No. 1, Chongqing, China

**Keywords:** finite-element analysis, biomechanics, posterior endoscopic cervical discectomy, radiculopathy, safety

There was a mistake in Figure 6 as published. In Figure 6, the labeling on the X-axis contains an error. In the beginning, we named it T0-T8 (T refer to tunnel), but now, we call it M0-M8 (M refer to model). The corrected Figure 6 appears below.

**FIGURE 6:**
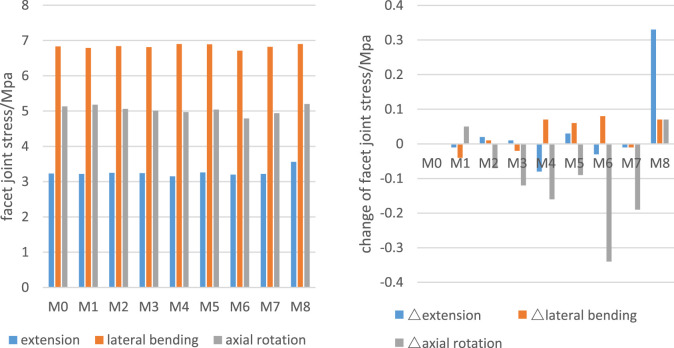
C6 facet joint stress and changes of the number.

There was a mistake in Figure 7 as published. In Figure 7, certain Chinese characters are present, which should be replaced with their English equivalents for clarity and consistency. The corrected Figure 7 appears below.

**FIGURE 7:**
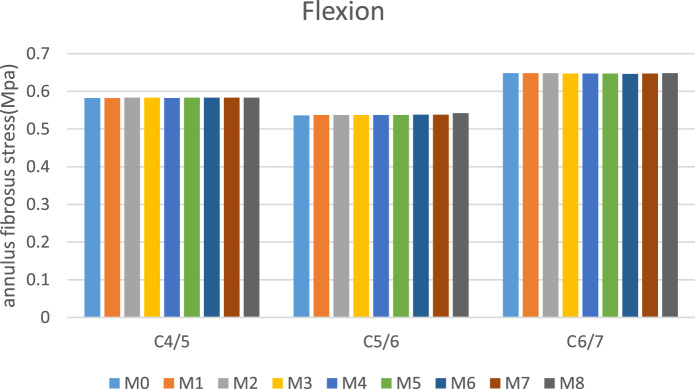
C5/6 nucleus pulposus stress and annulus fibrosus stress.

